# Account of an Extra Uterine Conception

**Published:** 1791

**Authors:** William Baynham

**Affiliations:** Member of the Corporation of Surgeons of London; and Surgeon in Essex County in Virginia.


V. *Account of an Extra-uterine Conception. Communicated in a Letter to Dr.* Simmons *by Mr.* William Baynham, *Member of the Corporation of Surgeons of London, and Surgeon in Effex County in Virginia.*

ABOUT ten years ago Mrs. Cock, the wife of a refpectable planter in this ftate, became pregnant a third time, and at the proper time was feized with labour pains, which continued for a day or two, and then left her. She remained in a weak and declining ftate for fome months after; during which time fhe was vifited and attended by feveral medical practitioners, two of whom declared her difeafe to be a dropfy of the uterus. At length, however, fhe regained her health, flefh, and ftrength, and fuffered only a trifling inconvenience from her increafed fize, which was equal, when I firft faw her, to that of a woman in the feventh month of pregnancy. In this ftate I found her foon after my return to this my native country; and at my firft interview with her I told her I was firmly perfuaded that fhe

ſhe had actually been with child, but that the child had never been in the womb; in which opinion I was more and more confirmed in every ſubſequent converſation with her.

Some months ago, after a ſevere attack of the influenza, which has for the laſt two years raged here with very great, and, during the laſt fall, with fatal violence, the abdominal tumour began to be painful, and was accompanied with a ſlight redneſs and inflammation of the ſkin, near to, and a little on the left of the navel. After attending her for ſome conſiderable time, during which I made one attempt, but failed, to relieve her by extracting the child, I was induced, by circumſtances, to undertake the operation a ſecond time; which I accordingly performed on Saturday laſt.

I made an inciſion in the belly, beginning oppoſite to, and a little on the left of the navel, and carrying it a finger's breadth or two obliquely downwards, and to the right towards the linea alba, I continued it afterwards in a ſtraight direction, cloſe to the left of the linea alba, about half way to the os pubis. Through this I, with ſome difficulty, extracted the child by pieces; from the appearances of which I judge it to have been equal in ſize (when whole) to a full-grown fœtus of nine months.

Some degree of putrefaction had taken place in in the child, ſo as to denude the greater part of the bones of the perioſteum and other coverings; but ſome of the ſoft parts ſtill retained their colour and texture, particularly the heart and lungs, which were perfectly freſh and ſound, and are in my poſſeſſion, preſerved in ſpirits.

I could find no remains of a navel ſtring or placenta, although both muſt have exiſted; but they had probably rotted, and come away in the diſcharge of matter which had come on, and continued a few weeks previouſly to the operation, through a ſmall opening that remained after my firſt attempt. Although the fœtus could not have been ſupported without the intervention of a navel ſtring and a vaſcular chorion, yet it will perhaps admit of a doubt whether or not the ſpongy ſubſtance, as in a common placenta, had an exiſtence.

The particulars of the caſe at large I mean to draw up at my leiſure, with the addition of ſome obſervations, which I will tranſmit to you, together with a ſpecimen of the bones. Mean time I flatter myſelf you will not be ſorry at receiving this abridged account of ſo very uncommon a caſe.

I left my patient yeſterday, being the fourth day after the operation, as well as could be expected; and my horſe is now waiting at the door to carry me thirty miles to ſee her again to-day to-day—which latter circumſtance I offer as an apology for the haſte in which I write; as before my return home the ſhip will have ſailed by which you will receive this letter.

*Eſſex County,*

*Rappahannock River,*

*Virginia,*

January 18, 1791.

